# Tumor Proteins D52 and D54 Have Opposite Effects on the Terminal Differentiation of Chondrocytes

**DOI:** 10.1155/2017/6014278

**Published:** 2017-07-17

**Authors:** Chihiro Ito, Yoshiki Mukudai, Masakatsu Itose, Kosuke Kato, Hiromi Motohashi, Toshikazu Shimane, Seiji Kondo, Tatsuo Shirota

**Affiliations:** ^1^Department of Oral and Maxillofacial Surgery, School of Dentistry, Showa University, 2-1-1 Kitasenzoku, Ota-ku, Tokyo 145-8515, Japan; ^2^Department of Biochemistry, School of Dentistry, Showa University, 1-5-8 Hatanodai, Shinagawa-ku, Tokyo 142-8555, Japan; ^3^Department of Oral and Maxillofacial Surgery, Faculty of Medicine, Fukuoka University, 7-45-1 Nanakuma, Jonan-ku, Fukuoka 814-0180, Japan

## Abstract

The tumor protein D (TPD) family consists of four members, TPD52, TPD53, TPD54, and TPD55. The physiological roles of these genes in normal tissues, including epidermal and mesenchymal tissues, have rarely been reported. Herein, we examined the expression of TPD52 and TPD54 genes in cartilage in vivo and in vitro and investigated their involvement in the proliferation and differentiation of chondrocytes in vitro. TPD52 and TPD54 were uniformly expressed in articular cartilage and trabecular bone and were scarcely expressed in the epiphyseal growth plate. In MC3T3E-1 cells, the expressions of TPD52 and TPD54 were increased in a differentiation-dependent manner. In contrast, their expressions were decreased in ATDC5 cells. In ATDC5 cells, overexpression of TPD52 decreased alkaline phosphatase (ALPase) activity, while knock-down of TPD52 showed little effect. In contrast, overexpression of TPD54 enhanced ALPase activity, Ca^2+^ deposition, and the expressions of type X collagen and ALPase genes, while knock-down of TPD54 reduced them. The results revealed that TPD52 inhibits and that TPD54 promotes the terminal differentiation of a chondrocyte cell line. As such, we report for the first time the important roles of TPD52 and TPD54, which work oppositely, in the terminal differentiation of chondrocytes during endochondral ossification.

## 1. Introduction

The tumor protein D52 (TPD52) family (reviewed in [1, 2]) consists of four members, that is, TPD52, TPD53 (known as TPD52L1) [3–6], TPD54 (TPD52L2) [5, 6], and TPD55 (TPD52L3) [7]. Of these, TPD52 was the first to be identified nearly 20 years ago as an overexpressed gene in breast cancers [8]; the gene is located on chromosome 8q21, a region frequently gained in various human cancers [9–11]. Thereafter, other members have also been reported to be highly expressed in various cancers, including prostate [12, 13], testis [7, 14], colon [15, 16], ovary [17–19], breast [20–22], and oral [23, 24] cancer cells. As such, it appears that TPD52 family proteins might play important roles in the invasion, growth, and metastasis of cancer cells. Furthermore, we recently reported that TPD52 and TPD54 have opposite effect in oral squamous cell carcinoma-derived cell lines [23, 24]. However, to the best of our knowledge, the expressional distributions and physiological roles of TPD52 family genes in normal tissues, including epidermal and mesenchymal ones, have rarely been reported.

The vertebrate skeleton develops via two separate mechanisms: intramembranous ossification and endochondral ossification. The flat bones of the skull, parts of the craniofacial skeleton, and the clavicles are formed by intramembranous ossification, and the rest of the craniofacial bones and the axial and appendicular skeleton are generated by endochondral ossification (reviewed in [25]). In both processes, the first step is condensation of mesenchymal progenitor cells at the site of the future bones. In intramembranous ossification, the condensed cells directly differentiate into bone-producing osteoblasts (reviewed in [26]). In contrast, in endochondral ossification, a cartilaginous template is later replaced by bone. Cartilage elements grow from the proliferation of chondrocytes with a type II collagen-rich extracellular matrix (ECM); these chondrocytes differentiate into prehypertrophic chondrocytes that produce increased levels of type X collagen and alkaline phosphatase (ALPase) when they finally undergo apoptosis [25]. Every step of endochondral ossification is regulated by the concerted actions of various growth factors, signaling molecules, and cytokines, such as type I, II, and X collagens, ECM components (e.g., osteonectin, osteocalcin, osteopontin, fibronectin, and CD44), and early response genes, such as* c-fos* [27].

In the present study, we focused on the tissue-specific distribution of the TPD52 and TPD54 in bone and cartilage tissue in vivo and the cell-specific physiological roles of those proteins on proliferation and differentiation of chondrocytes in endochondral ossification in vitro. As a result, we found that TPD52 and TPD54 regulate the hypertrophy of chondrocytes in opposite ways and that they might play important roles in the terminal differentiation of chondrocytes in cartilage tissue during endochondral ossification.

## 2. Materials and Methods

### 2.1. Cell Cultures

MC3T3-E1 cells (a mouse osteoblastic cell line) were cultured in *α*-minimum essential medium (MEM; Wako, Osaka, Japan) supplemented with 10% fetal bovine serum (FBS). ATDC5 cells (a mouse chondrosarcoma cell line) were cultured in Dulbecco's modified Eagle's medium nutrient mixture F-12 Ham (DMEM/F-1; Wako) supplemented with 10% FBS. RAW264.7 cells (a mouse monocyte and precursor of macrophage cell line) were cultured in RPMI 1640 medium (Wako) supplemented with 10% FBS. All cells were grown at 37°C in 5% CO_2_ and 100% humidity. For induction of differentiation, Osteoblast-Inducer Reagent (Takara Bio, Shiga, Japan) for MC3T3E1 cells, Insulin-Transferrin-Sodium Selenite Supplement (Roche Diagnostics, Mannheim, Germany) for ATDC5 cells, and a combination of 10 ng/ml of recombinant human receptor activator of nuclear factor kappa-B ligand (RANKL; Wako) and 10 ng/mL of recombinant human macrophage colony-stimulating factor (M-CSF; R&D, Minneapolis, MN, USA) for RAW264.7 cells were added to the cultures.

### 2.2. Immunohistochemistry

The sacrifice of the experimental animal complied with the Showa University Guidelines for Animal Experiments, and the experimental protocol was approved by the Animal Experimentation Committee of Showa University (approval number 28D009). A 12-week-old Slc:SD rat (Sankyo Labo Service, Tokyo, Japan) was sacrificed, and the tibias were removed and fixed in 10% buffered neutral formalin (Wako). The fixed specimens were decalcified with Kalkitox (Wako) and embedded in paraffin. Then, the sections were cut on a rotary microtome, were mounted on microscope slides, and were subjected to Hematoxylin-Eosin (H-E) staining as described previously [28]. Thereafter, the sections were incubated overnight at 4°C in a humid chamber with primary antibodies: anti-TPD52 antibody (1/100 dilution; Santa Cruz, Dallas, TX, USA) or anti-TPD54 antibody (1/200 dilution; Proteintech, Rosemont, IL, USA). On the next day, sections were incubated with the secondary antibody (EnVision+ System-HRP Labelled Polymer Anti-Rabbit; Dako, Carpinteria, CA, USA). Sections were reacted with the Dako Liquid DAB+ Substrate Chromogen System (Dako) and examined under a microscope, and photographs were taken.

### 2.3. Protein Preparation and Western Blot Analysis

Total cellular protein was prepared as described previously [29], and the protein concentration was measured using Quick Start Bradford Reagent (Bio-Rad, Hercules, CA, USA). Twenty micrograms of protein was subjected to sodium dodecyl sulfate polyacrylamide electrophoresis (SDS-PAGE) in a 4 to 20% gradient gel (Bio-Rad), and the blot was transferred onto a polyvinylidene difluoride membrane by iBlot 2 (Life Technologies, Carlsbad, CA, USA). After blocking with 0.2% nonfat dry milk (Cell Signaling Technology, Danvers, MA, USA) in Tris-buffered saline (Takara Bio), the membrane was incubated with the primary antibodies (anti-TPD52 antibody (1/1,000 dilution; Abcam, Branford, CT, USA), anti-TPD54 antibody (1/1,000 dilution), or anti-glyceraldehyde 3-phosphate dehydrogenase (GAPDH) antibody (1/10,000 dilution; Sigma-Aldrich, St. Louis, MO, USA) and the horseradish-peroxidase-conjugated secondary antibody (GE Healthcare UK Ltd., Buckinghamshire, UK)) as described previously [29]. The protein bands were visualized using Amersham ECL Western Blotting Detection Reagents (GE Healthcare) and a Chemidoc XRS Plus ImageLab System (Bio-Rad).

### 2.4. RNA Purification and RT-qPCR

The cells were seeded at a density of 10,000 cells/well in a 12-well tissue culture plate and cultured for 7 days. Total cellular RNA was purified using TRIzol (Life Technologies) according to the manufacturer's protocol. Ten nanograms of total RNA was reverse-transcribed using a commercial kit (iScript cDNA Synthesis Kit; Bio-Rad), and an aliquot of the reaction mixture (1/20) was used for the subsequent qPCR reaction. qPCR was carried out with a KAPA SYBR FAST qPCR kit (Kapa Biosystems, Boston, MA, USA), and statistical analysis was performed using Bio-Rad iQ5 analysis software (Bio-Rad). The fold changes in gene expression were calculated using the 2^−ΔΔCt^ method. The gene expression levels were first normalized to GAPDH within each sample group. All of the primer sequences for RT-qPCR are shown in Table 1.

### 2.5. Molecular Constructs, Small Interfering RNAs (siRNAs), and Transfection

The coding regions of mouse TPD52 and TPD54 cDNAs were amplified by an RT-PCR technique using single-stranded cDNA reverse-transcribed from total RNA of ATDC5 cells as a template. The sequences of the primer pairs for the coding regions of mouse TPD52 and TPD54 are shown in Table 2. The sense and antisense primers harbor* Bgl* I and* Kpn* I sites, respectively, and the amplicons were double-digested with the enzymes and were inserted into the corresponding site of pCMV-HA (Clontech, Mountain View, CA, USA) as described previously [24]. Proper constructs were confirmed by nucleotide sequencing using an ABI PRISM 310 Genetic Analyzer (Applied Biosystems, Foster City, CA, USA) and a BigDye Terminator v3.1 Cycle Sequencing Kit (Applied Biosystems). Control siRNA and siRNAs for mouse TPD52 and TPD54 genes were purchased from Sigma-Aldrich. The vectors and siRNAs were transfected into cells in a 6-well tissue culture plate using Lipofectamine 2000 (Life Technologies) according to the manufacturer's protocol.

### 2.6. Biochemical Assays

The transfected cells were reseeded at a density of 1,000 cells/well in a 48-well tissue culture plate and cultured for 7 days. Thereafter, the cells were lysed with 0.3 ml of 0.02% Triton X-100 (Sigma-Aldrich) in a physiological saline. DNA content, ALPase activity, sulfated glycosaminoglycan (GAG), and calcium (Ca^2+^) deposition were measured as described previously [30, 31].

### 2.7. Cytohistochemistry

Cells were seeded at a density of 1,000 cells/well in 48-well tissue culture plates and allowed to grow to maturation for 7 days. Then, the cells were stained for ALPase activity by crystal violet and toluidine blue staining as described previously [30, 31].

### 2.8. Statistical Analysis

Unless otherwise specified, all experiments were repeated at least three times, and similar results were obtained in the repeated experiments. Statistical analysis of the repeatability of the assay results was carried out using an unpaired Student's* t*-test. Data are expressed as means ± standard deviation of triplicate data. Significance was determined at ^*∗*^*p* < 0.05.

## 3. Results

### 3.1. Expression of TPD52 and TPD54 Proteins in Bone and Cartilage In Vivo and In Vitro

**Figure 1:**
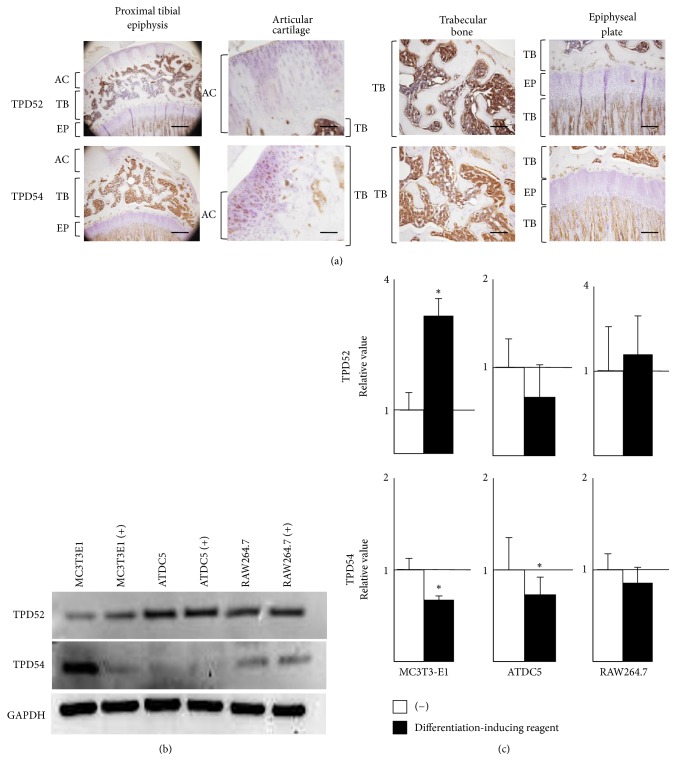
Assessment of TPD52 family expression in vivo and in vitro. (a) Immunohistochemistry. A 12-week-old rat was sacrificed and the tibias were removed and subjected to immunohistochemistry for TPD52 and TPD54. Bar, 100 *μ*m. (b) Expression of TPD52 and TPD54 proteins in mouse cell lines. MC3T3-E1, ATDC5, and RAW264.7 cells were cultured in the absence (−) or presence (+) of a differentiation-inducing reagent (see Materials and Methods) and were subjected to western blot analysis for TPD52, TPD54, and GAPDH. (c) Expression of TPD52 and TPD54 genes in mouse cell lines. MC3T3-E1, ATDC5, and RAW264.7 cells were cultured in the absence (open box) or presence (closed box) of a differentiation-inducing reagent (see Materials and Methods) and were subjected to RT-qPCR for TDP52 and TPD54. The value of the uninduced cells was designated as “1,” and relative values are shown. Data are shown as the mean with the standard deviation of three sets of cultures. ^*∗*^*p* < 0.05 versus the control.

First, we examined the expressions of TPD52 and TPD54 in the proximal tibial epiphysis of a 12-week-old rat by immunohistochemical staining (Figure 1(a)). TPD52 and TPD54 were uniformly expressed in articular cartilage and trabecular bone but were scarcely expressed in the epiphyseal plate. Based on these in vivo results, the expressions of TPD52 and TPD54 proteins and genes were examined in three cell lines, that is, MC3T3-E1, ATDC5, and RAW264.7 with or without differentiation-inducing supplementation, by western blot analysis (Figure 1(b)) and RT-qPCR (Figure 1(c)). In MC3T3-E1 cells, the expression of the TPD52 gene was increased by an osteogenic stimulation, whereas the expressions of the TPD54 gene and protein were decreased by that stimulation. In contrast, in ATDC5 cells, mRNA levels of TPD52 and TPD54 were decreased by the differentiation-inducing stimulation. In RAW264.7 cells, TPD52 and TPD54 were uniformly expressed regardless of the osteoclast-inducing stimulation. These results showed that TPD52 family proteins might be involved in the proliferation and/or differentiation of osteoblasts and chondrocytes, but not of osteoclasts.

### 3.2. TPD52 and TPD54 Have Little Involvement in the Proliferation and Differentiation of Osteoblasts

**Figure 2:**
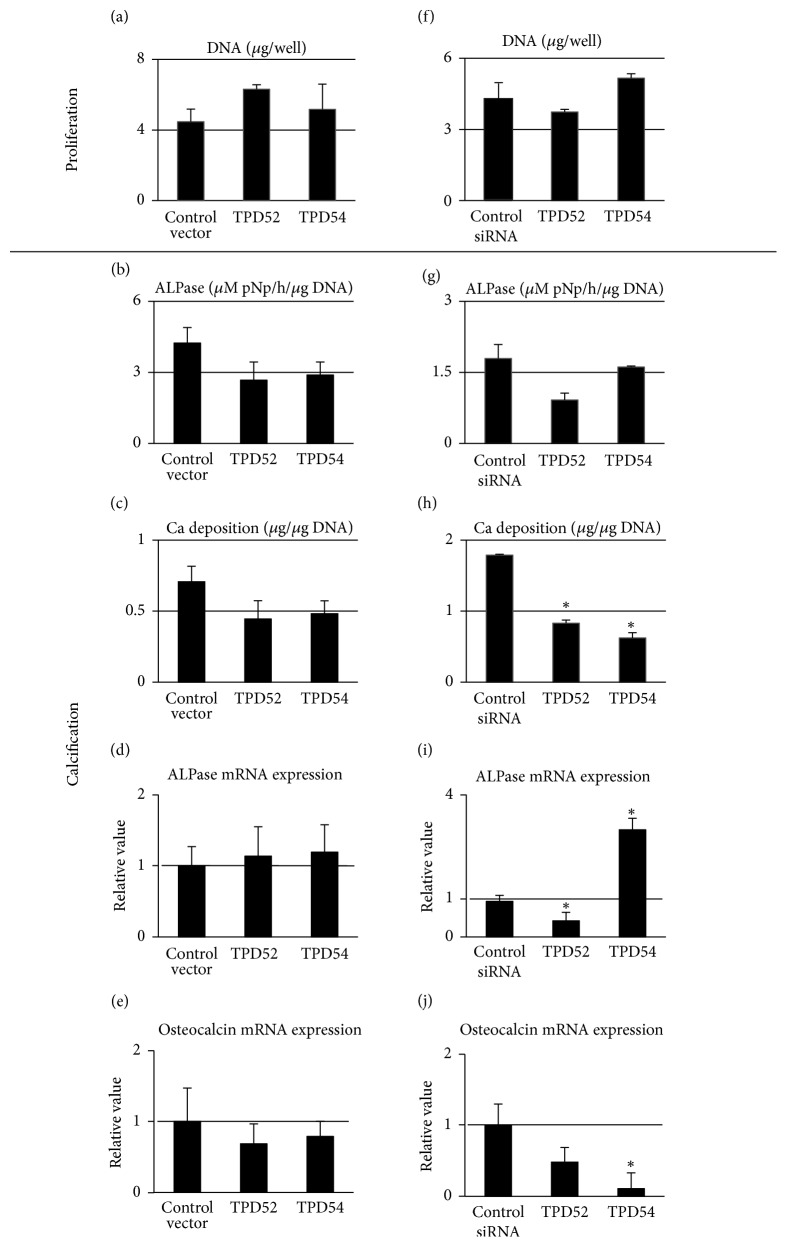
Analysis of the overexpression or knock-down of TPD52 and TPD54 in MC3T3-E1 cells. The pCMV-HA control (control vector), HA-tagged TPD52 and TPD54 overexpression vectors (a to e) or control, and TPD52 and TPD54 siRNAs (f to j) were transfected into MC3T3-E1 cells, and the cells were subjected to DNA (a and f), ALPase activity (b and g), and Ca deposition (c and h) measurements, as well as RT-qPCR for ALPase (d and i) and osteocalcin (e and j). Data are shown as the mean with the standard deviation of three sets of cultures. ^*∗*^*p* < 0.05 versus the control.

Since the results of the previous subsection led to a hypothesis that the TPD52 and TPD54 genes might play an important role in the proliferation and/or differentiation of osteoblasts and chondrocytes, we initially investigated these effects in MC3T3E1 cells by inducing the overexpression or knock-down of TPD52 and TPD54 using cytomegalovirus- (CMV-) promoter-driven-hemagglutinin- (HA-) tagged expression vectors and small interfering RNAs (siRNAs), respectively, followed by cell-biological assays and RT-qPCR (Figure 2). Neither overexpression nor knock-down of the genes affected cell proliferation (Figures 2(a) and 2(f)). The overexpression (Figures 2(b)to 2(e)) of TPD52 and TPD54 showed little effect (*p* > 0.05) on the differentiation of the cells. Knock-down of TPD54 induced mRNA expression of ALPase significantly (Figure 2(i)), while the increasing effect on ALPase activity was little (Figure 2(g)). Also, knock-down of TPD52 decreased mRNA expression of ALPase (Figure 2(i)). The opposite results were observed for Ca^2+^ deposition and osteocalcin mRNA expression (Figures 2(h) and 2(j)). These results led us to conclude that TPD52 and TPD54 genes might have little involvement in the proliferation and differentiation of osteoblasts, although more detailed experiments remained.

### 3.3. TPD52 and TPD54 Play More Important Roles in the Terminal Differentiation of Chondrocytes Than in Prehypertrophic Maturation

**Figure 3:**
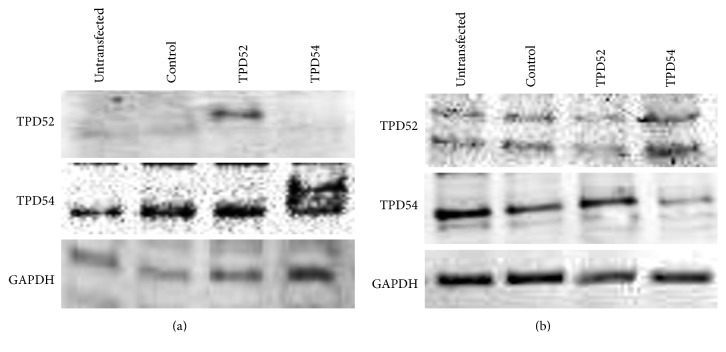
The overexpression and knock-down of TPD52 and TPD54 in ATDC5 cells. The pCMV-HA empty vector (Control), HA-tagged TPD52 and TPD54 overexpression vectors (a) or control, and TPD52 and TPD54 siRNA (b) were transfected into ATDC5 cells. After 48 h, the total proteins of the transfected and untransfected cells (untransfected) were purified and subjected to western blot analysis for TPD52, TPD54, and GAPDH (an internal control).

**Figure 4:**
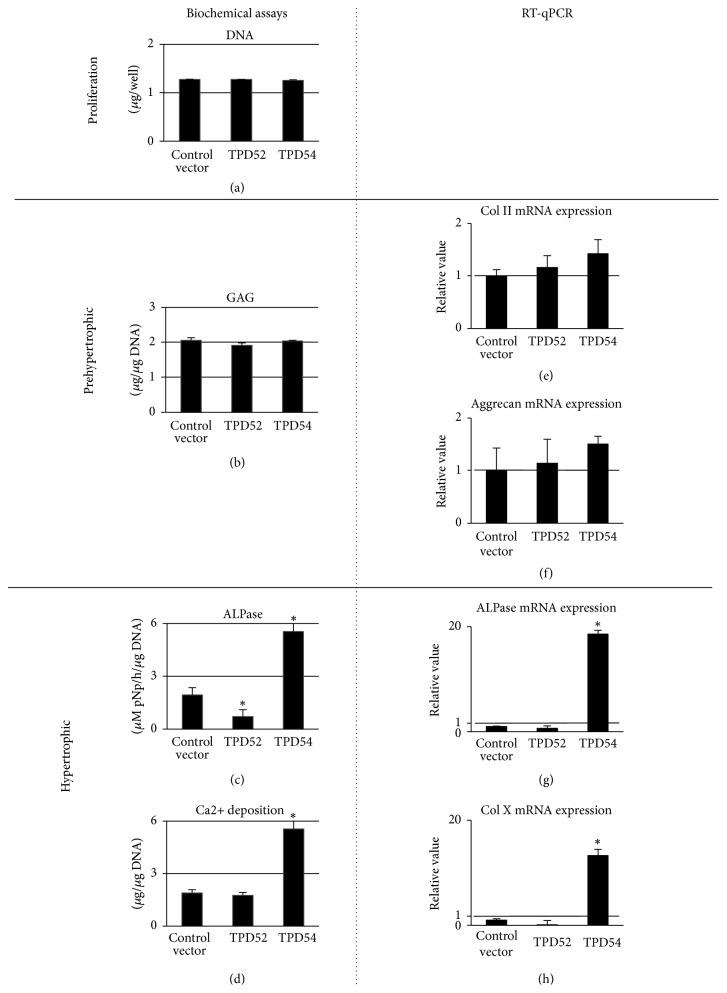
Analysis of the overexpression of TPD52 and TPD54 in ATDC5 cells. The pCMV-HA control (control vector) and HA-tagged TPD52 and TPD54 overexpression vectors were transfected into ATDC5 cells, and the cells were subjected to DNA (a), GAG (b), ALPase activity (c), and Ca deposition (d) measurements, as well as RT-qPCR for type II collagen (e), aggrecan core protein (f), ALPase (g), and type X collagen (h). For RT-qPCR, the value of the control was designated as “1,” and relative values are shown. Data are shown as the mean with the standard deviation of three sets of cultures. ^*∗*^*p* < 0.05 versus the control.

**Figure 5:**
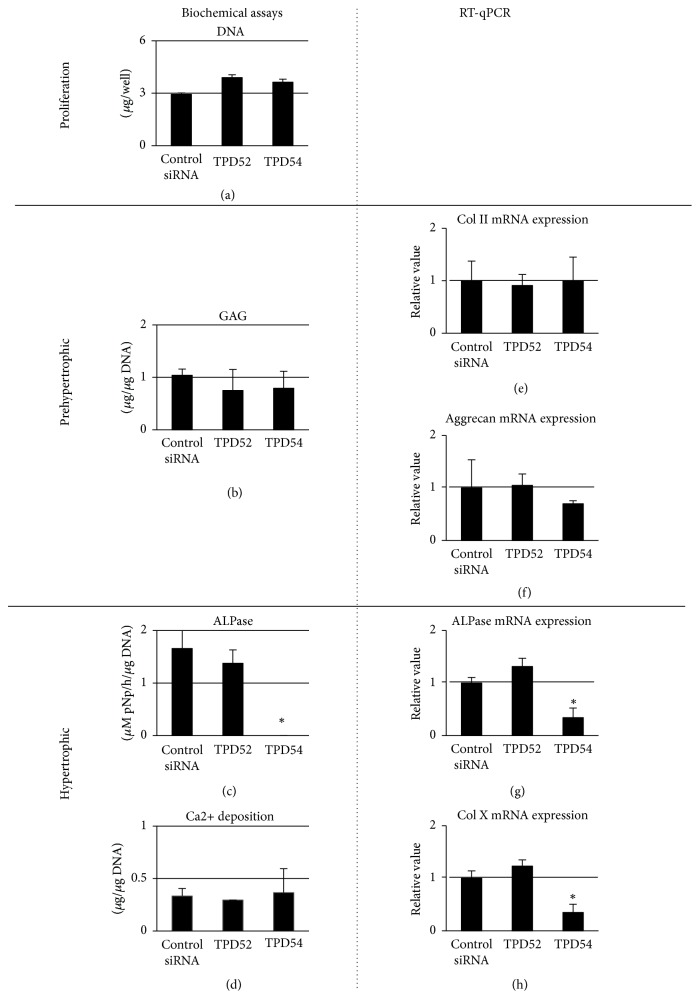
Analysis of the knock-down of TPD52 and TPD54 in ATDC5 cells. Control, TPD52, and TPD54 siRNAs were transfected into ATDC5 cells, and the cells were subjected to DNA (a), GAG (b), ALPase activity (c), Ca deposition (d), measurements, as well as RT-qPCR for type II collagen (e), aggrecan core protein (f), ALPase (g), and type X collagen (h). For RT-qPCR, the value of the control was designated as “1,” and relative values are shown. Data are shown as the mean with the standard deviation of three sets of cultures. ^*∗*^*p* < 0.05 versus the control.

**Figure 6:**
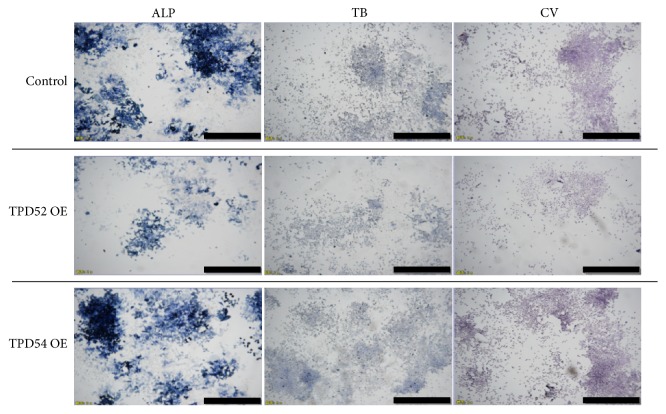
Cytohistochemical analysis of TPD52 and TPD54 overexpression and knock-down in ATDC5 cells. The pCMV-HA overexpression vectors (OE) and siRNAs (KD) for TPD52 and TPD54 were transfected into ATDC5 cells. After 7 days, the cells were subjected to ALPase (ALP), crystal violet (CV), and toluidine blue (TB) staining. Bar, 1 mm.

Next, we investigated the effects of TPD52 and TPD54 genes on the proliferation and differentiation of ATDC5 cells.  Figure 3 shows the successful transfection of HA-tagged TPD52 and TPD54, and of TPD52 and TPD54 siRNAs, resulting in both the overexpression and knock-down of these genes. We focused on roles of those proteins on proliferation and differentiation of chondrocytes in endochondral ossification and investigated the effects of TPD52 and TPD54 genes on the proliferation and differentiation of ATDC5 cells. We investigated the biological effects of the overexpression (Figure 4) and knock-down (Figure 5) of these genes in ATDC5 cells. Neither the overexpression nor the knock-down of these genes showed an effect on cell proliferation (Figures 4(a) and 5(a)). The overexpression of TPD52 decreased ALPase activity (Figure 4(c)), whereas the overexpression of TPD54 enhanced ALPase activity and its gene expression (Figures 4(c) and 4(g)), as well as Ca2+ deposition (Figure 4(d)) and type X collagen gene expression (Figure 4(h)). On the other hand, knock-down of TPD52 showed less effects (Figures 5(b)to 5(h)), and of note, knock-down of TPD54 significantly reduced ALPase activity and its gene expression (Figures 5(c) and 5(g)), as well as type X collagen gene expression (Figure 5(h)). However, the maturation markers of prehypertrophic chondrocytes (i.e., type II collagen and aggrecan) were barely altered by the overexpression or knock-down of the two genes (Figures 4(b), 4(e), 4(f), 5(b), 5(e), and 5(f)). Similar results were observed in cytohistochemistry (Figure 6). The overexpression of TPD52 decreased ALPase activity, and the knock-down of TPD54 did not show any modulating effect on that decrease. On the other hand, the overexpression of TPD54 increased ALPase activity. In addition, only small effects were seen on toluidine blue or crystal violet staining.

## 4. Discussion

TPD55 was found to be limited to normal testis [7]. On the other hand, the other members (TPD52, TPD53, and TPD54) have been reported to be highly expressed in various cancers. We recently reported that TPD52 and TPD54 have opposite effect in oral squamous cell carcinoma-derived cell lines [23, 24]. However, the expressional distributions and physiological roles of TPD52 family genes in normal tissues, including epidermal and mesenchymal ones, are not well understood. Moreover, there are no reports on roles of these proteins in endochondral ossification. Therefore, in the present study, we sought to identify the physiological roles of TPD52 and TPD54 proteins in cartilage metabolism and revealed for the first time in the world that TPD52 and TPD54 are proteins that are involved in hypertrophy.

Ummanni et al. [32] reported that TPD52 expression activates Akt/phosphoinositide-3 kinase (PI3K) signaling, and we recently [24] demonstrated that TPD54 is a negative regulator of Akt/PI3K signaling and decreases the migration and adhesion of oral squamous cell carcinoma cells. In chondrocytes, Akt/PI3K signaling is required for physiological hypertrophic cell differentiation and endochondral bone growth [33], whereas Kita et al. [34] reported that the Akt/PI3K pathway blocks the terminal differentiation of chondrocytes. In addition, p21^cip1^ and p27^waf1^, which are located downstream of the Akt/PI3K pathway (reviewed in [35]), are negative regulators of the proliferative zone in chondrogenesis and lead to apoptosis in preparation for ossification [36]. The present study suggested that TPD52 expression might activate the Akt/PI3K signaling pathway, blocking the terminal differentiation of chondrocytes; this leads to the hypothesis that TPD52 might enhance cell proliferation through the Akt/PI3K signaling pathway and maintain the prehypertrophic state of chondrocytes. On the other hand, TPD54 might terminate the prehypertrophic state of chondrocytes and initiate the start of terminal differentiation. As such, the expressions of TPD52 and TPD54 might concordantly play an important role in the regulation of the terminal differentiation of chondrocytes. It is also suggested that some kind of cartilaginous abnormality might occur if the balance between TPD52 and TPD54 expressions collapses.

Taken together, the results from the present study showed that TPD52 and TPD54 play an important role in terminal differentiation of chondrocytes with opposite effects on the terminal differentiation during endochondral ossification. In order to address the points, more detailed investigations using the knock-out mouse are ongoing; we will be able to report in the future.

## 5. Conclusions

The present study revealed that overexpression of TPD52 inhibits the terminal differentiation phenotypes for prehypertrophic chondrocyte, whereas overexpression of TPD54 promotes them. However, overexpressions of those genes showed little effects on proliferation and maturating phenotypes for proliferating chondrocytes. Consequently, we report for the first time the important roles of TPD52 and TPD54, which work oppositely, in the terminal differentiation of chondrocytes during endochondral ossification.

## Figures and Tables

**Table 1:** Primers used in RT-qPCR. The primer sequences for the sense and antisense strands used in the RT-qPCR are shown. Col II*α*1, type II collagen *α*1 chain; Col X*α*1, type X collagen *α*1 chain; ALPase, alkaline phosphatase.

Col II*α*1	Sense	ACTGGTAAGTGGGGCAAGAC
	Antisense	CCACACCAAATTCCTGTTCA
Col X*α*1	Sense	CTCCTACCACGTGCATGTGAA
	Antisense	ACTCCCTGAAGCCTGATCCA
Aggrecan	Sense	AGGACCTGGTAGTGCGAGTG
	Antisense	GCGTGTGGCGAAGAA
ALPase	Sense	TGACCTTCTCTCCTCCATCC
	Antisense	CTTCCTGGGAGTCTCATCCT
GAPDH	Sense	TGACGTGCCGCGTGGAGAA
	Antisense	AGTGTAGCCAACATGCCCTTCAG
TPD 52	Sense	ATGGAGTGCAGAGATATGGA
	Antisense	TCAGGGGCTCTCTGTCATCTGT
TPD54	Sense	ATGGACTCTGCTAGCCAAGA
	Antisense	TTAGAAAGGCGCATGATCCGGC

**Table 2:** Primers used for molecular constructs. The sequences of primer pairs for the coding regions of mouse TPD52 and 54 are shown.

TPD 52	Sense	GAAGATCTCTATGGAGTGCAGAGATATGGA
	Antisense	GGGGTACCTCAGGGGCTCTCTGTCATCTGT
TPD54	Sense	GAAGATCTCTATGGACTCTGCTAGCCAAGA
	Antisense	GGGGTACCTTAGAAAGGCGCATGATCCGGC
